# Self-Organising Map Based Framework for Investigating Accounts Suspected of Money Laundering

**DOI:** 10.3389/frai.2021.761925

**Published:** 2021-12-14

**Authors:** Abdallah Alshantti, Adil Rasheed

**Affiliations:** ^1^ Department of Engineering Cybernetics, Norwegian University of Science and Technology, Trondheim, Norway; ^2^ Department of Mathematics and Cybernetics, SINTEF Digital, Trondheim, Norway

**Keywords:** self-organising map (SOM), money laundering, suspicious accounts, risk levels, investigation strategies

## Abstract

There has been an emerging interest by financial institutions to develop advanced systems that can help enhance their anti-money laundering (AML) programmes. In this study, we present a self-organising map (SOM) based approach to predict which bank accounts are possibly involved in money laundering cases, given their financial transaction histories. Our method takes advantage of the competitive and adaptive properties of SOM to represent the accounts in a lower-dimensional space. Subsequently, categorising the SOM and the accounts into money laundering risk levels and proposing investigative strategies enables us to measure the classification performance. Our results indicate that our framework is well capable of identifying suspicious accounts already investigated by our partner bank, using both proposed investigation strategies. We further validate our model by analysing the performance when modifying different parameters in our dataset.

## 1 Introduction

Money laundering represents a major challenge for governments and financial institutions alike, as the flow of dirty money can hinder a state’s development, damage the reputation of the financial system and motivate the generation of further crime (Kumar, 2012). Since money laundering is an underground market, its true magnitude can never be precisely determined. However, the most recent estimate of the laundered money worldwide considers the sum of illicit transactions worldwide to account for 2–5% of the global GDP (Levi and Reuter, 2006). In addition, financial institutions that fail to deploy satisfactory measures for investigating and reporting suspicious cases, thus not sufficiently filing suspicious activity reports, are subject to heavy fines and penalties by the authorities. Therefore, there has been an increasing interest over the past decades to develop tools that aid in the combat against money laundering.

The most common methods used by financial institutions for detecting and reporting suspicious cases are rule-based systems. A rule-based system is a set of predefined rules gathered from a knowledge base maintained by domain experts. Transactions that match the predefined conditions trigger alerts, which prompt further investigations by bank compliance teams. A major drawback of the rule-based systems is the generation of a significant volume of false positive alerts that are costly in terms of time and resources needed to track down flagged cases (Gao, 2009). Those false positive alarms are estimated to constitute more than 90% of the total alerts generated by the traditional rule-based systems commonly adopted by banks (Breslow et al., 2017). Consequently, there has been an increasing desire to develop more advanced tools for more precise detection of money laundering transactions.

In several studies, new frameworks and systems were developed for identifying money laundering transactions or accounts (Liu et al., 2008; Le-Khac et al., 2009; Lopez-Rojas and Axelsson, 2012; Jun, 2006; Soltani et al., 2016). While many of these works claim a good detection performance, they often use synthetic data due to the limited availability of financial transactional data. Therefore, the reliability of such methods was not tested on real financial data, which might indicate that there might be no practical use for such frameworks. In addition, money laundering datasets are heavily characterised by a significant class imbalance, where the number of normal transactions tremendously exceeds the number of illicit transactions. The class imbalance represents a major challenge in the detection of suspicious transactions, and as a result, the data is often either undersampled or oversampled to improve the classification performance (Sudjianto et al., 2010). Another significant challenge is the quality of the data and the labels used. It is estimated that sum of money from the money laundering cases investigated annually make up no more than 10% of the total amount laundered (Argentiero et al., 2008). Therefore, while financial institutions may have labelled datasets that indicate which transactions were investigated as suspicious, it is highly likely that these labels represent only a fraction of the total number of money laundering transactions that went undetected.

Overall, the most prevalent limitation in AML literature is that many of the proposed models are developed with the aim of replacing human intervention (Chen et al., 2014). In essence, these studies aim to detect money laundering transactions in a binary classification setting. While machine learning based automation is increasingly deemed as a valuable mean in AML for tasks such as transaction monitoring (Turki et al., 2020) and processing client information (Tiwari et al., 2020), a sensitive field such as AML cannot solely rely on machine learning algorithms to replace human expertise for reporting illegal transactions. Instead, compliance teams have an obligation of providing their reasoning when filing suspicious activity reports (Singh and Lin, 2020). The requirement to include the explanation when submitting suspected money laundering cases to the authorities entails that the adoption of technologies in the finance compliance sector ought to aid the decision making of case investigators, rather than fully automating the money laundering detection process.

In this work we aim to address these underlying gaps in literature by proposing a SOM-based approach to assist in the decision making process of compliance teams when investigating bank accounts. A SOM is an unsupervised neural network that maps a highly dimensional dataset into a lower-dimensional representation (Kohonen, 2012). SOMs are commonly used in clustering (Isa et al., 2009; Haga et al., 2015; Nilashi et al., 2020), classification (Yorek et al., 2016; Jain et al., 2018) and oversampling (Douzas and Bacao, 2017) applications. For its notable success in these applications, we propose a SOM based method for clustering and classifying bank accounts into risk levels that can be further investigated by the bank. Consequently, our implementation of SOM is a hybrid approach that combines the strengths of unsupervised and supervised learning. In contrast to traditional clustering techniques where the parameter selection significantly impacts the grouping of observations, the number of clusters in SOMs is dependent on the number of training observations. The main parameters we introduce contribute to the categorisation of the formed clusters, such that these parameters can be tuned according to how strict or lenient the decision makers preferences are. Moreover, unlike K-means clustering, the dimension reduction in SOM helps in visualising how the bank accounts are distributed across the low-dimensional space, which can be used for further inferring how the accounts are grouped together.

To this end, we develop a novel framework based on SOMs to categorise the bank accounts in our dataset into different risk level groups. First, the data is pre-processed and aggregated to group the transactions by account. This is an important step since grouping the data by accounts enables the model to capture the historic financial patterns exhibited by the accounts. The most important features in the dataset are selected and the dataset is split into a training and test sets in a k-fold cross-validation manner. We then select the SOM and its hyperparameters and develop a categorisation technique for dividing the SOM neurons and the accounts assigned to them into three risk level groups based on the neurons’ properties. The model’s performance is evaluated by proposing two investigation strategies using different utilisations of the risk levels. Finally, we further evaluate the classification performance of our model by varying the number of selected features and the ratio of suspicious accounts in the data.

Taking the limitations of existing AML research and our motivation for this work into consideration, we highlight our contributions as follows:

1) We develop a framework that facilitates the investigation of potential money laundering accounts despite the major class imbalance between the normal and suspicious accounts in the dataset. The SOM model maps the observations into a 2-dimensional grid space, and in doing so, the technique attempts to group the unlabelled observations with similar transaction patterns together.
2) We contribute to the state of the art by introducing a system that can well handle poorly labelled financial transaction data. Since the training of our SOM grid does not rely on the whether a given sample is suspicious or not, our method takes into account the challenges of assigning the transactions with their true labels by financial compliance teams. This is due to the fact that money laundering transactions are often carried out in a way that makes them difficult to distinguish from normal transactions (Teichmann, 2020).

The remainder of this paper is divided as follows. In Section 3, we review the existing literature on anti-money laundering and self-organising maps. We describe our dataset and the preprocessing method in Section 4. Section 5 discusses the methodology which consists of feature selection, SOM architecture and risk level categorisation. In Section 6, we evaluate the performance of investigation strategies and present the results of the training and test sets. The paper is concluded and motivations for future work are provided in Section 7.

## 2 Literature Review

### 2.1 Anti-Money Laundering

Several existing studies on money laundering detection have been focusing on data mining and supervised machine learning techniques when the data provided contains the labels of the previously identified transactions. (Liu et al., 2008) developed a sequence matching algorithm to identify suspicious sequences in an account’s history by comparing the similarities and differences with other sequences in the data. Similarly, (Tang and Yin, 2005) proposed a one-class SVM algorithm to identify suspicious and unusual patterns while highlighting the speed and efficiency of their method. (Jullum et al., 2020) trained an XGBoost supervised model using financial transactional data for predicting whether certain financial transactions should be reported, and demonstrated that their method outperforms the existing approach used by financial institutions. The detection of suspicious transactions linked to terrorism financing was implemented by (Rocha-Salazar et al., 2021) using real datasets provided by a financial institution in Mexico, which was found to reduce the number of false positives in comparison to rule-based system flagging.

Meanwhile, unsupervised methods are employed when the labels are unavailable or when using synthetic datasets. (Wang and Dong, 2009) proposed a minimum spanning tree clustering method to detect money laundering transactions using different tree levels. In a study by (Chen et al., 2014), clustering of transactions to identify the suspicious transactions grouped together was carried out using an expectation maximisation algorithm. A distance based clustering technique was combined with a local outlier detection method to identify suspicious transactions in a synthetic dataset (Gao, 2009). (Paula et al., 2016) developed an unsupervised deep learning algorithm based on an autoencoder to detect anomalous transactions in Brazilian exports financial data.

In addition, several works follow AML approaches that focus on identifying accounts and customers rather than transactions. Social network analysis was used for identifying the roles and responsibilities of criminals in money laundering networks (Dreżewski et al., 2015), and for analysing overall group properties of criminal networks (Savage et al., 2016). Moreover, (Zhou et al., 2017) developed a statistical classifier for the detection of suspicious accounts involved in illicit virtual currency trade, while achieving a low rate of false positives in their approach. While (Wang and Yang, 2007) implemented decision trees based approach to determine the money laundering risk levels of customers of a commercial bank, only the customers’ profiles were used to fit the model without taking into account their transactional data.

In this work, we combine the benefits of supervised and unsupervised learning to cluster and categorise our bank’s clients into risk level groups. It is noteworthy to recognise that in the works mentioned above, the low proportion of money laundering transaction was mainly resolved by oversampling or undersampling prior to the implementation of the methods. In our work, we instead use datasets with several class ratios to demonstrate that our approach is reasonably effective on both heavily imbalanced datasets and balanced datasets. Additionally, we present an adaptive approach that considers the level of suspicion at an account level instead of analysing every transaction individually. This is necessary, since a significant proportion of money laundering transactions have very similar characteristics to ordinary transactions. The consideration of an account’s history in an aggregated manner allows the inclusion of historic patterns that could be linked to money laundering behaviour. Moreover, the aforementioned studies presented models that were aimed to replace human expertise by attempting to replicate their decisions, which is essentially problematic due to the challenges with manually identifying money laundering transactions. Instead, we propose a method that ranks the suspicious level of accounts in order to assist compliance teams at financial institutions with prioritising the clients to further investigate.

### Self-Organising Maps

A self-organising map is an unsupervised neural network that maps a multi-dimensional dataset along a lower-dimensional grid (Kohonen, 1990). Due to their structural properties, SOMs have been widely implemented for different use-cases in various industries. (Yorek et al., 2016) combined SOM with ward clustering to classify living organisms into three distinguished clusters. SOM for clustering was also used for classifying natural language written texts into their respective document types (Pacella et al., 2016). Furthermore, (Kiang et al., 2006) applied SOM on telecommunication questionnaire responses in order to cluster the respondents into several segments by using K-means clustering. (Lacerda and Mello, 2013) presented a framework that segments handwritten digits using SOM for accurate digit recognition.

In addition to clustering, self-organising maps have been increasingly employed for oversampling the minority class’s observations when a class imbalance exists (Kim, 2007; Cai et al., 2014; Douzas and Bacao, 2017). Whereas, (Zadeh Shirazi et al., 2018) developed a framework using SOM and complex-valued neural network for the diagnosis and detection of breast cancer using a dataset consisting of five features for hundreds of patients. Meanwhile, (Stephanakis et al., 2019) proposed an extensive SOM-based hybrid method that was used for anomaly detection in cloud computing structures. (Barreto, 2007) established that unlike other artificial neural network models, SOM-based models can be used for time-series prediction whilst benefiting from SOM’s interpretable results.

Furthermore, SOMs have also been explored in the financial domain. In a study by (Du Jardin and Séverin, 2011), SOM was used to forecast the financial models of companies over extended periods of time and demonstrated a better prediction accuracy than traditional methods. SOMs were also employed for predicting bankruptcy by analysing financial records of several companies (Kiviluoto, 1998). Meanwhile, (Hsu, 2011) presented a hybrid approach using self-organising maps and genetic programming for predicting stock closing prices. In our work, we draw inspiration from such studies to demonstrate the capability of a SOM-based model in identifying potential money launderers, in contrast to the traditional supervised learning methods which rely heavily on the assigned labels for classification. The employment of SOM in our study stems from the need for grouping of accounts with similar transactional attributes together, in order for the investigators to able to make sense of these clusters. The clustering is followed by the categorisation of the lower dimensional grid to assign a risk level for every client in our dataset which are subsequently used for measuring the performance of the proposed investigation strategies.

## 3 Data

In this work, we use the financial transactional data provided to us by our partner bank, DNB, the largest financial group in Norway. The data made available to us for this study represents a fraction of the total financial transactions handled by the bank between January 2014 and December 2016, with the bank clients as the main party and either bank clients or external accounts as the second party. The data has already been labelled by the bank, such that a class label exists for each transaction as to whether the transaction is normal or suspicious. In this context, suspicious transactions are not the alerts generated by the rule-based system, but are the more serious cases which were carefully investigated by the DNB’s compliance team as potential money laundering cases - most of which were reported to the financial authorities. Subsequently, the labels in our dataset do not reveal whether the suspicious cases were indeed money laundering cases, since these decisions are made separately by the financial authorities and their outcome is not made available to us in the provided dataset. To this end, we treat every transaction that was thoroughly investigated by the compliance team at the bank as a suspicious transaction.

### 3.1 Aggregation

In practice, it is almost impossible to make a decision purely based on transaction features of an individual transaction when investigating a particular case. Investigators often look at history of the party involved to observe if there are any underlying patterns or changes in a client’s financial activity (Singh and Best, 2019). In this work, we relatively follow the investigators’ approach by aggregating the financial transactions data on the accounts. The original transactional dataset consists of a combination of categorical, numerical and mixed type features. We refine the dataset to eliminate the redundant and duplicate features. The outline of the refined dataset is presented in Table 1.

**TABLE 1:** Dataset attributes prior to aggregation.

Attribute type	Number of attributes	Attribute names	Unique categories
Identifier	1	Account number	
Date	1	Transaction date	
Numerical	1	Transaction amount	
Categorical	9	CreditDebitCode	2
		TransTypeID	29
		TransMethodID	21
		TransactionChannelID	28
		TextCode	84
		ProductCode	292
		CurrencyCodeOrig	112
		SourceSystemOrg	110
		SourceSystemFetch	3
Binary	1	Class label	

The only identifier variable we select is the account number of the first party for every transaction in the dataset. To aggregate the data by account, we first apply one-hot encoding to the categorical variables. This increases the number of categorical variables from 9 variables to 681 variables. During the process of aggregation, these categorical variables are converted to the frequency of occurrence of every one-hot encoded feature for every account’s transaction history between 2014 and 2016. The final feature is the binary label feature that indicates whether an account had a transaction that was investigated as a potential money laundering transaction. For accounts without any suspicious cases, all the transactional data was used in the aggregation process. Accounts that were involved in suspicious transaction had their data aggregated from their first transaction until the last suspicious transaction. This was done for two reasons. First, in some cases banks allow their customers to carry on with their financial activity while an investigation is pending. Second, for suspicious customers we are mainly interested in the transactions that led to the investigations, therefore, we are not concerned with the transaction activity that took place afterwards.

### 3.2 Dataset Size

When aggregating by accounts, we decide to eliminate bank accounts with more than 50,000 transactions over a 3 year period from the data. This is supported by the fact that accounts used by large corporations are quite distinct from the majority of personal and corporate accounts. As such, outlying behaviour was removed to maintain the focus on the classification of bank accounts of customers with average account usage.

The ratio of money laundering transactions is tremendously low compared to the normal transactions. As our partner bank, DNB, would not like to disclose the true ratio of suspicious clients in the dataset, we set the ratio of suspicious accounts in the dataset at % 10 of the total accounts in our baseline model. A subset of the suspicious accounts from our data is chosen such that 1,141 suspicious accounts are represented in the dataset. The remaining 10,269 accounts in the baseline model are ordinary accounts that were not investigated for money laundering by the bank.

### 3.3 Preprocessing

Additional features are generated in order to embed a combination of the most significant attributes for every account. For every observation we engineer a total of 17 features, which include attributes such as the average number of daily credited transactions, average amount per transaction and the cumulative sum of debited transaction. The engineered features are first normalised by taking the natural logarithm of their values +1. We then normalise all the engineered features in order for the values to fall in the [0,1] scale. Therefore, after dropping the date and the identifier attributes, all the features in the preprocessed dataset become in the [0,1] range, as the one-hot encoded ratios features are already within the same scale. We then drop all the single-valued features for all the 11,410 observations. Subsequently, we end up with 522 features after dropping all the non-unique features.

### 3.4 Training and Test Sets

We divide our baseline dataset which consists of 11,410 accounts and 522 features into training and test sets. In the split, we use a 80/20 training/test set ratio in a 5-fold cross-validation. Additionally, we ensure the ratio of suspicious customers is exactly 10% in both the training and test sets. The labels are initially removed from both the training and test sets and are reattached afterwards for evaluation. Later in our work, we measure the performance of our method using the mean value of the classification metrics of the 5-fold cross-validations, and a confusion matrix obtained by adding the predicted and actual labels of the test samples from all the cross-validation runs.

## 4 Methodology

**FIGURE 1:**
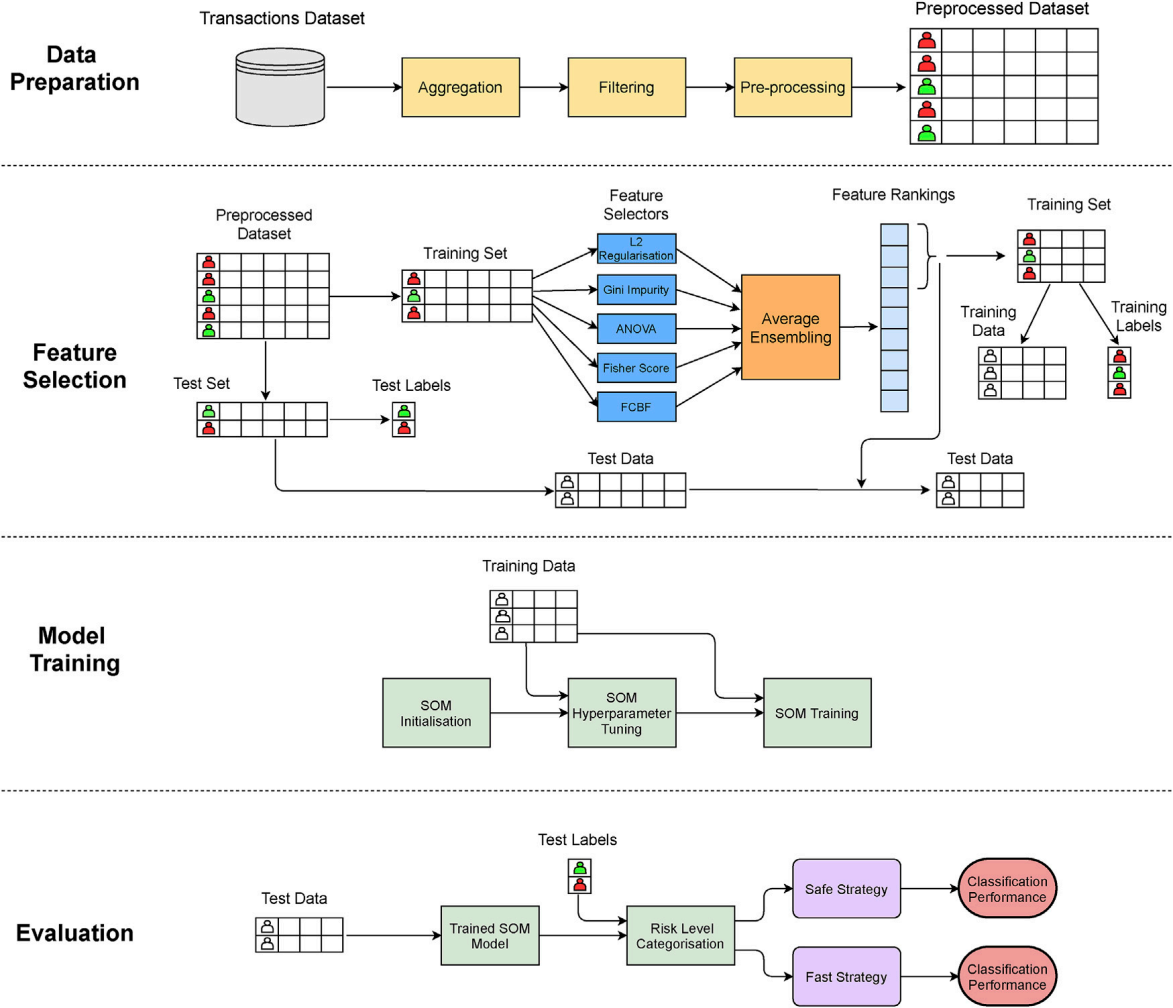
Architecture of the SOM money laundering classification framework.

In our work, we implement a 4-stage approach in order to predict whether every bank client in our dataset might be involved in a money laundering case or not. We use the preprocessed dataset described in Section 4 for fitting and evaluating the presented model. Figure 1 depicts an overview of the stages of our proposed method.

### 4.1 Feature Selection

We use an ensemble of feature selection algorithms in order to select the most significant features of the training set contributing to the label of the observations. This is important, as the dimensionality of the dataset can be reduced by discarding the noisy and redundant variables (Guyon et al., 2008). Five feature selection algorithms are implemented on the dataset which rank the features according to their importance. Since the feature selection algorithms rank the features differently, we use an ensemble of the feature selectors. The ensemble computes the average ranking of every feature from the five feature selection methods, and subsequently, the features are ranked according to their mean rankings.

#### 4.1.1 L2 Regularisation

Regularisation is a technique used in machine learning to increase the training error in order to improve the generalisation on unseen data by preventing the overfitting of data. In L2 regularisation, also known as ridge regularisation, a loss function is computed using the weight coefficients of features and a bias term. The weights are updated using gradient descent optimisation to minimise the loss function. Since the labels are binary, the error function is also the log loss function used in logistic regression to predict the labels. When the regularisation converges, the features with greater weights are considered as the more important ones in the dataset.

#### 4.1.2 Gini Impurity

The Gini impurity for feature importance is computed using the random forest classifier (Breiman, 2001). A random forest is an ensemble of decision trees, where each tree is a set of nodes and leaves. In classification problems, a given node in a decision tree is characterised by a feature with a certain threshold to decide how to split the dataset into two sets based on how the observations over and below the feature threshold are attributed to the different classes. The Gini impurity for each feature calculates the importance as the sum over the number of splits among the trees comprising the feature, proportionally to the number of samples the feature splits.

#### 4.1.3 ANOVA F-Score

Analysis of Variance, ANOVA, is a univariate F-test that compares the mean of a feature between two or more label classes. The univariance property implies that the means and variances for a single feature against the target class are computed at a given time. The null hypothesis is that a feature’s population means is the same for all the different labels. The method is commonly used as a statistical feature selection to measure how well individual features in a dataset can contribute to the target class labels.

#### 4.1.4 Fisher Score

The Fisher score is a similarity-based feature selection method that computes the feature importance, and is typically used in binary classification applications (Hart et al., 2001). The algorithm works by calculating the Laplacian matrix for a given feature using the local affinity and the global affinity matrices for all the observations and their labels. The Fisher score is then calculated using the inverse of the Laplacian score generated from the Laplacian matrix. Higher scores are awarded to features that well separate instances of different classes from each other, while drawing the observations of the same class closer together.

#### 4.1.5 FCBF

Fast Correlation-Based Filter Solution, FCBF, is a mutlivariate feature selection technique developed by (Yu and Liu, 2003) that calculates the importance of features by estimating the interdependencies between them. The dependencies between a set of features can be calculated using a Symmetrical Uncertainty (SU) value based on information gain and entropy measures. In this fast feature selection approach, features which are given a higher score, implying more importance, are those which are highly correlated to the class labels, but are less correlated to other features.

### 4.2 Self-Organising Map

A SOM is an unsupervised neural network algorithm developed by (Kohonen, 1990), where a high dimension dataset is typically mapped into a two dimensional representation arranged in either a rectangular or a hexagonal topology. Extensions to the traditional SOM include the time-adaptive self-organising map (Shah-Hosseini and Safabakhsh, 2003), which is a dynamic implementation of SOM that updates the learning parameters as more datapoints are added or modified over a period of time. However, due to the scarcity of observations and the class imbalance in our dataset, we implement a framework based on the traditional SOM instead of the time-adaptive self-organising maps. After selecting only the most relevant features in the dataset as highlighted in Section 5.1, the training labels are detached from the dataset, such that the training of the SOM model is carried out without the class labels.

#### 4.2.1 SOM Description

The SOM consists of two layers: the prototype input layer and the output layer. The number of neurons in the output layer are determined by selecting the respective dimension parameters when creating the SOM. Each neuron is represented by a high-dimensional vector in the input layer, where the dimension size of each prototype vector is equivalent to the number of features in the data. The training of the SOM algorithm is implemented by the steps described below:

##### 1 Initialisation

The weights of the input prototype vector is initialised. This is done by either assigning the weights randomly, sampling observations from the data as the weights or by using linear methods such as the first two principal components of the principal component analysis for assigning the initial weights to the prototype vector.

##### 2 Choosing a Random Sample

An observation from the dataset is chosen at random for training the weights of the SOM layers.

##### 3 Matching

The best matching unit (BMU) is found by computing the Euclidean distance between the observation and the prototype vectors corresponding to the neurons in the output space. The prototype vector that is the closest to the observation, denoted by the minimum distance, will assign its neuron in the outer layer as the BMU. This is described by:

$$∥x_{j}-m_{c}∥\;=\min_{i}∥x_{j}-m_{i}∥$$ (1)

where *x*_*j*_ is the observation vector, *c* is the index of the BMU, *m*_*c*_ is the prototype vector of the BMU, *i* is the number of neurons, and *m*_*i*_ is the prototype vector corresponding to neuron *i*.

##### 4 Neighbourhood Calculation

The neighbouring neurons of the BMU are determined. In the first stages of the training, the neurons are relatively close to each other. The distance between neurons increases over time, thus the number of neighbours decreases.

##### 5 Weight Updating

In this stage, the BMU is rewarded by closely matching the observation vector. Neighbouring neurons are also matched to the observation sample, but to a lesser extent. The SOM update rule for the prototype vector *i* is:

$$m_{i}\left(t+1\right)=m_{i}\left(t\right)+\alpha \left(t\right)\;h_{ci}\;\left(x_{j}\left(t\right)-m_{i}\left(t\right)\right)$$ (2)

where *t* is the time step, *α* is the learning rate at time *t*, and *h*_*ci*_ is the neighbourhood function. The learning rate parameter is selected when choosing the model hyperparameters and fall within the [0,1] range. The neighbourhood function weights the neighbourhood kernel around the BMU in the output map and is usually in the form of a Gaussian function. The neighbourhood function at a given time step can be calculated as:

$$h_{ci}\left(t\right)=\exp\left(-\frac{∥r_{c}-r_{i}{∥}^{2}}{2\sigma^{2}\left(t\right)}\right)$$ (3)

where *r*_*c*_ is the position vector of the BMU neuron, *r*_*i*_ is the position vector of the other neurons in the SOM output map, and *σ*(*t*) is the neighbourhood spread radius function at time *t*.

##### 6 Iteration

Steps 2–5 are repeated based on the number of iterations specified before training the SOM algorithm.

#### 4.2.2 SOM Hyperparameter Selection

The dimensions of the SOM grids are selected according to $5\sqrt{N_{tr}}$ rule proposed by (Vesanto and Alhoniemi, 2000), where *N*_*tr*_ is the number of training observation samples used for SOM training. The dataset used in the baseline model consists of 11,410 observations, 80% of which are used in each cross-validation training, hence 9,128 samples. This gives us $5\sqrt{9128}=477.70$ neurons. Applying the square root and rounding to closest integer gives us 22 neurons in each dimension, thus, a 22 × 22 SOM grid for the baseline model.

We choose a hexagonal topology for the SOM grid implementation since each non-edge neuron is surrounded by six neurons, rather than four neighbouring neurons commonly computed in the rectangular topology. In addition, we choose a Gaussian function for updating the neurons’ prototype weights since the Gaussian function allows all the neurons to be updated, not just the ones in close proximity to the BMU. For the learning rate and the neighbourhood spread radius function, we tune these hyperparameters on the training set prior to training the SOMs by calculating the quantisation error of the grid over a range of values for the two parameters. Since we train our model using 5-fold cross validations, we train five different SOMs, each with its unique learning rate and neighbourhood spread parameters based on the quantisation error generated from the training data in every cross-validation run.

### 4.3 Categorisation

Following the model training, the neurons in the SOM grid are categorised into three risk levels based on their distance from neurons in the neighbourhood, which we refer to as the inter-neural distance, and their suspicious ratio composition. For a given neuron, the inter-neural distance is the normalised sum of Euclidean distances between the neuron’s weight vector, *m*_*i*_ from Eq. 2, and its neighbouring weight vectors when the model converges. Neurons with larger inter-neural distances are well separated from their neighbours, and therefore have more distinct properties. On the other hand, neurons with lower inter-neural distances have similar properties to their neighbouring neurons. Since the inter-neural distances of all SOM neurons fall in the [0,1] range, we divide this scale into five equally sized segments, *P*_1_, …, *P*_5_ for categorising the neuron’s risk levels:

$$\begin{array}{c} P_{1}=\left[0,0.2\right)=\left\{d_{i}\in P_{1}\;|\;0\leq d_{i}<0.2\right\}\\ P_{2}=\left[0.2,0.4\right)=\left\{d_{i}\in P_{2}\;|\;0.2\leq d_{i}<0.4\right\}\\ P_{3}=\left[0.4,0.6\right)=\left\{d_{i}\in P_{3}\;|\;0.4\leq d_{i}<0.6\right\}\\ P_{4}=\left[0.6,0.8\right)=\left\{d_{i}\in P_{4}\;|\;0.6\leq d_{i}<0.8\right\}\\ P_{5}=\left[0.8,1\right]=\left\{d_{i}\in P_{5}\;|\;0.8\leq d_{i}\leq 1\right\} \end{array}$$ (4)

where *d*_*i*_ is the inter-neural distance of neuron *i*.

As discussed earlier, each training observation is assigned to its BMU. The suspicious composition of a SOM neuron is the proportion of suspicious observations from the total number of observation assigned to them following the reattachment of labels after training. This indicates that neurons with a high suspicious composition tend to mainly comprise suspicious observations, hence can be seen as highly suspicious neurons themselves. Meanwhile, neurons with a low suspicious composition are treated as nodes that mainly encapsulate normal accounts. In contrast to the inter-neural distance, we divide the suspicious composition [0,1] range into three segments, *Q*_1_, *Q*_2_, *Q*_3_, that vary in size. The segments are defined as follows:

$$\begin{array}{c} Q_{1}=\left[0,z\right)=\left\{k_{i}\in Q_{1}\;|\;0\leq k_{i}<z\right\}\\ Q_{2}=\left[z,z+\beta \right)=\left\{k_{i}\in Q_{2}\;|\;z\leq k_{i}<z+\beta \right\}\\ Q_{3}=\left[z+\beta ,1\right]=\left\{k_{i}\in Q_{3}\;|\;z+\beta \leq k_{i}\leq 1\right\} \end{array}$$ (5)

where *z* is the ratio of suspicious accounts in our dataset, *k*_*i*_ is the suspicious composition of neuron *i*, and *β* is the boundary threshold. In principle, the boundary threshold, *β*, can take the form of any value within [0,1]. However, we set *β* = 0.25 in our SOM implementation to ensure the neurons in *Q*_2_ have a comparable representation, similar to neurons in segments *Q*_1_ and *Q*_3_.

**FIGURE 2:**
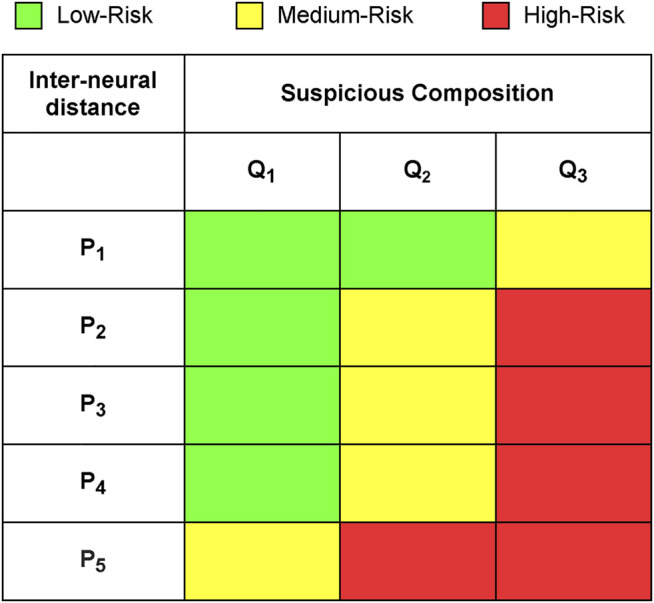
SOM neurons risk categorisation matrix.

The inter-neural distance segments and suspicious composition segments are used for constructing the neuron categorisation matrix, such that every neuron belongs to a risk level. We establish that neurons with large inter-neural distances and large suspicious compositions are very likely attributed to observations that pose a high risk for money laundering. Meanwhile, neurons that are in close proximity to their neighbours and generally have a smaller proportion of suspicious accounts are unlikely to encapsulate suspicious observations. Nodes with intermediate inter-neural distance and suspicious composition values are considered as medium-risk nodes. Our risk level categorisation of neurons based on the inter-neural distance and suspicious composition is demonstrated in Figure 2.

### 4.4 Strategy Selection

Samples are the given the same risk level as their best matching neuron. In this way, we can observe how the samples are distributed along the risk categorisation matrix, just like the SOM neurons. Therefore, a given observation can either belong to the low-risk, medium-risk or high-risk money laundering category. Since we have three risk level categories, and two target class labels: normal or suspicious, we propose two strategies to embed the risk categories into the binary labels of the accounts. These two strategies represent the different approaches that can be adopted by the banks or financial institutions in order to prioritise the investigation of accounts over a period of time based on the desired considerations of the risk groups. This is also instrumental for evaluating the performance of our model and highlighting the differences between the two approaches. The proposed two strategies are:

• *Safe Strategy*: Medium-risk and high-risk accounts are classified as suspicious.
• *Fast Strategy*: Only high-risk accounts are classified as suspicious.

In the safe strategy, the accounts that were categorised in the medium-risk and high-risk groups are considered as potentially suspicious and are to be investigated as such. In this strategy, banks are willing to act safely by investigating the transaction patterns of accounts falling in the medium and high risk groups. In contrast, in the fast strategy banks prioritise only the accounts in the high risk group for investigations. This can decrease the volume of accounts to be investigated and the amount of time spent investigating them. We therefore use both strategies for evaluating the performance of our model, while comparing the metrics of both approaches.

## 5 Results

We implement our model in Python using the Minisom library (Vettigli, 2021) to generate the self-organising maps and using the SKLearn library (Pedregosa et al., 2011) to evaluate the performance of our proposed method. In this section, we present the results produced after the implementation of the baseline model, and we further evaluate the model’s performance when experimenting with our dataset’s structure.

### 5.1 Baseline Model

In the baseline model, 11,410 accounts are split into a 80/20 training/test split in a 5-fold cross-validation. The ratio of suspicious accounts in the dataset is 10*%*, and the same ratio is maintained in the training and test cross-validation sets. In every cross-validation run we use the ensemble of feature selectors on the training set to select the top 25 ranked features generated from the five feature selectors in our dataset and discard the remaining features. The most important features from the training set are also selected for test data in the cross-validations prior to evaluation. This implies that a different set of features is generated for the training and test sets during every run.

#### 5.1.1 Training Set Analysis

**FIGURE 3:**
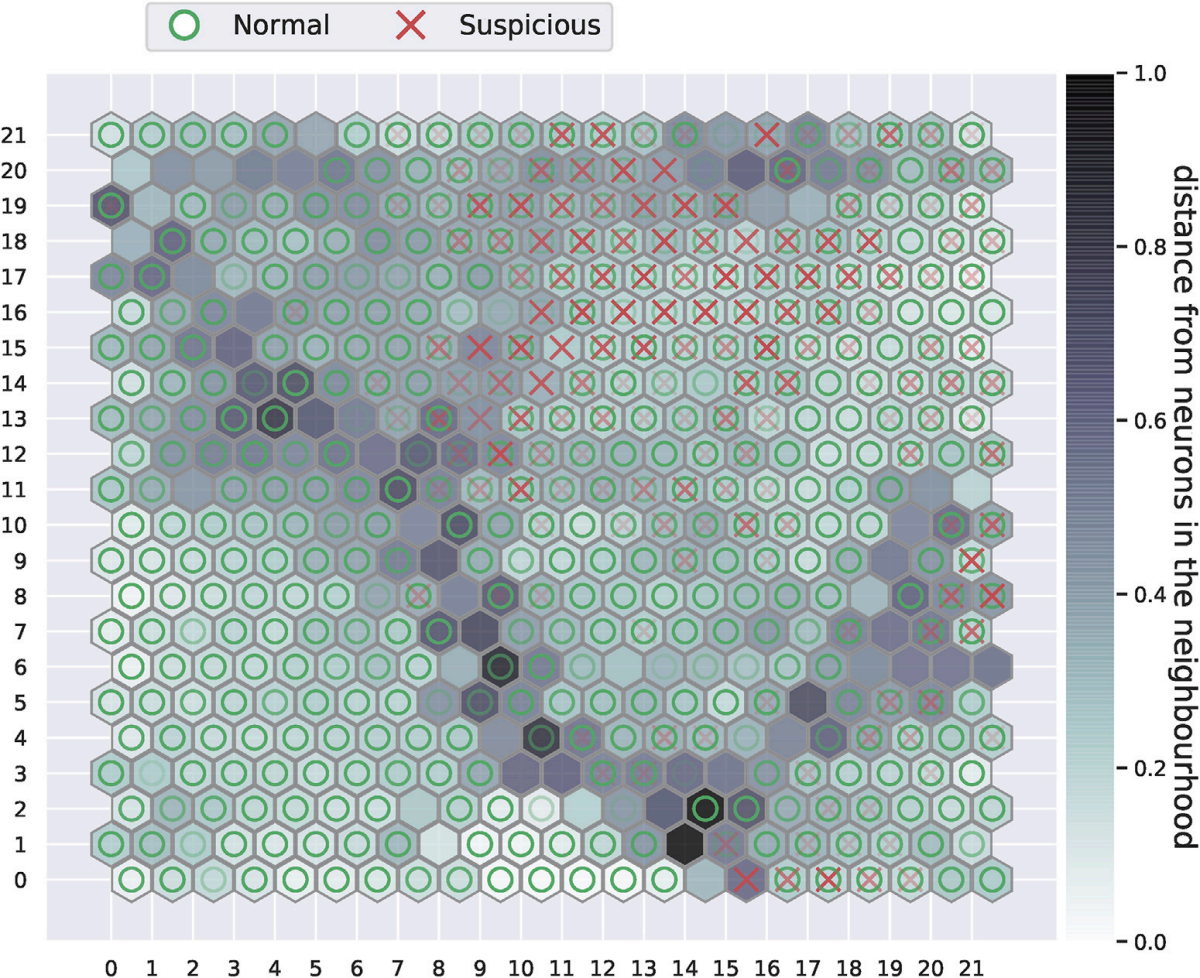
The self-organising map plot after training. Dark grey cells in the plot represent neurons that are further away from their neighbouring neurons, while the light grey or white cells are those that are in close proximity to their neighbours. Darker intensity markers of either the red crosses or green circles indicate the attribution of many observations of the same class to a given neuron.

To provide an insight on the performance of our method on the baseline training set, we present the training results of our fifth cross-validation run. In this manner, the SOM plot and the neurons distribution along the risk categorisation matrix from one of the iterations can be visualised. Figure 3 demonstrates the unsupervised SOM generated from the training set with 25 selected features.

It can be observed from the plot in Figure 3 that suspicious accounts, marked with red crosses in the plot, appear to accumulate towards the upper right side of the SOM. This is not exclusive to suspicious accounts, as other normal accounts can be seen attributed to the same neurons as the suspicious accounts. We can also observe how few suspicious observations are scattered around the grid. A distinct boundary that consists of neurons with large distances from each other can also be visualised from the self-organising map. These neurons have a large inter-neural distance, and they appear to divide the map into several regions. It can also be observed how a large region under the boundary on the lower left side is entirely composed of normal accounts. This can give an intuition that the some of the normal accounts have features that significantly separates them from suspicious accounts and other normal accounts. It is worth noting that the positioning of accounts, thus markers, vary between the training cross-validation runs since the training observations and the initialised weight vectors are changed. Nevertheless, the main properties of the SOM such as the dense suspicious regions and the partitions created by high inter-neural distance nodes are maintained throughout the cross-validations.

The SOM plot of the fifth cross-validation is used for categorising the neurons along the neurons risk categorisation matrix depicted in Figure 2. Since the baseline dataset comprises a 10% suspicious observations ratio, we use *z* = 0.10 for Eq. 5 to calculate the boundaries of the suspicious composition segments: *Q*_1_ = [0, 0.1), *Q*_2_ = [0.1, 0.35) and *Q*_3_ = [0.35, 1]. Subsequently, the neurons distribution along the risk categorisation matrix is provided in Table 2.

**TABLE 2:** Fifth-fold cross-validation SOM neurons inter-neural distances against their suspicious accounts composition.

Inter-neural distance	Suspicious composition			Total
	0–9.99%	10–34.99%	35+%	
<0.20	98	7	7	112
0.20–0.39	154	40	40	234
0.40–0.59	80	6	18	104
0.60–0.79	26	2	1	29
≥0.80	5	0	0	5
Total	363	55	66	484

From Table 2, it can be noted how the vast majority of the neurons have an inter-neural distance of less than 0.6. This implies that most neurons are in close vicinity to their neighbours, the only exception being the dark grey nodes in the SOM grid in Figure 3. Moreover, most neurons tend to have a suspicious composition of less than 10*%*. This is reasonable, given that the ratio of suspicious accounts in our dataset is 10*%*. Categorising the risk level of neurons according to the matrix in Figure 2 reveals that 365 neurons are low-risk neurons, 60 neurons are medium-risk and 59 neurons are high-risk. Given that observations are matched with the neurons in the SOM grid, we further present the distribution of training accounts in the categorisation matrix in Table 3.

**TABLE 3:** The distribution of the fifth-fold cross-validation training accounts along the risk categorisation matrix. Notation of the numbers is: all accounts (suspicious accounts).

Inter-neural distance	Suspicious composition			Total
	0–9.99%	10–34.99%	35+%	
<0.20	3122 (27)	119 (21)	158 (111)	3399 (159)
0.20–0.39	2973 (29)	602 (109)	647 (418)	4222 (556)
0.40–0.59	855 (9)	65 (11)	233 (164)	1,153 (184)
0.60–0.79	265 (4)	52 (7)	4 (2)	321 (13)
≥0.80	33 (0)	0 (0)	0 (0)	33 (0)
Total	7248 (69)	838 (148)	1,042 (695)	9,128 (912)

Similar to the neurons distributions, it can be observed in Table 3 how the majority accounts were attributed to neurons with low inter-neural distances and low suspicious compositions. We can see, however, that the majority of suspicious accounts were assigned to neurons with a large suspicious compositions. In other words, suspicious observations tend to be grouped together by the same neurons. Using this information, the risk grouping of training accounts is demonstrated in Table 4.

**TABLE 4:** Risk categorisation of the fifth-fold cross-validation training observations.

Risk category	All accounts	Suspicious accounts
Low	7334	90
Medium	910	238
High	884	584
Total	9,128	912

It can be evident from Table 3 how the training samples in the cross-validation run were categorised into the low, medium and high risk levels. We can see that while most of the non-suspicious accounts belonged to the low-risk group, 90 suspicious observations were misclassified as low-risk. In addition, the number of accounts in the low-risk category is significantly greater than total number of accounts in the medium and high-risk categories, which are comparable in size. The suspicious accounts, however, tend to be more concentrated in the high-risk category.

#### 5.1.2 Test Set Analysis

To evaluate the performance of our model on the test data, we combine the results from all five cross-validations, such that every sample in our dataset is used only once for testing. As such, we can represent the test data distribution on the risk categorisation matrix and the risk level grouping in Tables 5, 6 respectively.

**TABLE 5:** The distribution of test accounts along the risk categorisation matrix. Notation of the numbers is: all accounts (suspicious accounts).

Inter-neural distance	Suspicious composition			Total
	0–9.99%	10–34.99%	35+%	
<0.20	2379 (33)	94 (9)	72 (43)	2545 (85)
0.20–0.39	2809 (46)	513 (78)	407 (247)	3729 (371)
0.40–0.59	2548 (64)	513 (83)	534 (324)	3595 (471)
0.60–0.79	940 (12)	174 (37)	238 (128)	1,352 (177)
≥0.80	123 (5)	11 (2)	55 (30)	189 (37)
Total	8799 (160)	1,305 (209)	1,306 (772)	11,410 (1,141)

**TABLE 6:** Risk categorisation of test observations.

Risk category	All accounts	Suspicious accounts
Low	8770	164
Medium	1,395	246
High	1,245	731
Total	11,410	1,141

From Table 5 we can notice how the testing accounts are spread across the risk categorisation matrix. Similar to the training samples distribution, most of the accounts were assigned to BMUs with a low suspicious composition. An interesting observation, however, is that many accounts in the cross-validation test sets were assigned to neurons with inter-neural distances between 0.4 and 0.59, compared to the total training samples under the same inter-neural distance in Table 3. This entails that the SOM model performs a slightly different matching between samples and neurons when the model is learning from the input, compared to when the test data is applied to the trained model. The risk levels of the test accounts after all five cross-validations in Table 6 appear similar to the risk grouping of training accounts from Table 4. Most of the suspicious accounts were categorised as high-risk, while evidently smaller populations were assigned with low and medium-risk categories.

Our evaluation of the model relies on the investigation strategies proposed in Section 5.4, since we formulated three risk levels for the binary class labels in our dataset. We recall that in the safe strategy, the medium and high-risk accounts are classified as suspicious, whereas, the fast strategy only considers the high-risk accounts as suspicious. On that basis, we use the risk level categorisation of the test observations to construct a confusion matrix for the safe strategy and a confusion matrix for the fast strategy represented in Tables 7, 8, respectively.

**TABLE 7:** Safe strategy confusion matrix.

		Predicted	
		Normal	Suspicious
**Actual**	**Normal**	8606	1,663
	**Suspicious**	164	977

**TABLE 8:** Fast strategy confusion matrix.

		Predicted	
		Normal	Suspicious
**Actual**	**Normal**	9,637	632
	**Suspicious**	405	736

As it can be seen from the confusion matrices, the majority of the accounts that were classified as low-risk are in fact normal accounts. The difference between both strategies is more evident when looking at the number of false positives and the number of false negatives. The safe strategy is more conservative and detects more suspicious accounts. The drawback, however, is that 1,663 normal accounts were incorrectly classified as suspicious. In the fast strategy only 632 accounts were misclassified as suspicious, which is a drastic improvement in terms of reducing the number of false positives. However, this comes at the cost of obtaining more than twice the number of false negatives of the safe strategy. Table 9 presents the classification metrics for both strategies using the output of the confusion matrices.

**TABLE 9:** Classification performance metrics for the safe and fast strategies.

Strategy	Accuracy	Precision	Recall	F1-score	AUC
Safe	0.8399	0.3728	0.8562	0.5188	0.8472
Fast	0.9091	0.5480	0.6451	0.5897	0.7918

It can observed from Table 9 how the performance metrics differ for both strategies. The accuracy score is higher for the fast strategy, which is reflected by the large number of correctly classified normal accounts, or true negatives. The elimination of the medium-risk category in the fast strategy also improved the precision score for the fast strategy since the medium-risk category contained a substantial amount of normal observations. The precision scores for both strategies is nevertheless still low as several normal accounts have transactional features which are similar to suspicious accounts. As expected, the safe strategy has a better recall score than the fast risk investigation strategy. Classifying the accounts in the medium-risk category as suspicious enabled the detection of more suspicious accounts, thus the higher recall score for the safe strategy in comparison with the fast strategy. The calculated F1-score asserted equal weights to the recall and precision scores. Therefore, a greater F1-score is obtained by the fast strategy. Meanwhile, the receiver operating characteristic area under curve (AUC) scores are comparable for both approaches, but slightly higher for the safe strategy as a result of the increased rate of the true positive classification on various thresholds.

From the classification metrics, the precision-recall trade-off can be very well observed. A more conservative classification based on risk categories enables the detection of more suspicious accounts. However, this comes at the expense of investigating a larger number of normal accounts. The fast strategy is concerned with reducing the number of normal accounts to be investigated. However, this leads to missing out on some of the suspicious accounts that were categorised earlier in the medium-risk group. The F1-score can be tuned according to preference, if any, over the rate of false positives and the rate of false negatives.

### 5.2 Experimental Results

In principle, financial transaction datasets are heavily characterised by class imbalance where abnormal activity represents a small fraction of all transactions. As such, further experiments were carried out to analyse the model’s performance when changing the dataset’s structure. More precisely, it is interesting to study how the model behaves when tuning the ratio of suspicious accounts in our dataset. We run models on datasets with a suspicious class size of 5, 10, 20, and 50% of the total accounts in the datasets. To implement this, we select 500 suspicious observations to be used in all the experiments and modify the number of normal accounts according to the selected class ratios. Moreover, the number of selected features are tuned when training the SOMs, to investigate the impact of the number of features on the model’s ability to correctly classify the observations.

Given the two proposed investigation strategies, we identify the most important metrics for evaluating the performance as the recall score and the precision score. Although the F1-score is a useful metric for combining the recall and the precision performances, we instead use the recall and precision scores to assess to what extent the investigation strategies are capable of achieving their objective. Similar to the baseline model, every experiment was run in 5-fold cross-validations and the mean values of the recall and precision scores were calculated. These are presented in Tables 10, 11.

**TABLE 10:** Classification rate recall scores.

Suspicious ratio (%)	Safe strategy					Fast strategy				
	Number of Features					Number of Features				
	10	25	50	100	All	10	25	50	100	All
5	0.790	0.802	0.778	0.824	0.826	0.368	0.594	0.516	0.464	0.432
10	0.836	0.840	0.850	0.818	0.828	0.660	0.624	0.610	0.580	0.530
20	0.832	0.826	0.856	0.838	0.822	0.680	0.714	0.644	0.584	0.596
50	0.792	0.830	0.842	0.840	0.864	0.606	0.644	0.582	0.692	0.738

**TABLE 11:** Classification rate precision scores.

Suspicious ratio (%)	Safe strategy					Fast strategy				
	Number of Features					Number of Features				
	10	25	50	100	All	10	25	50	100	All
5	0.228	0.257	0.238	0.190	0.160	0.402	0.467	0.445	0.297	0.262
10	0.391	0.392	0.355	0.323	0.314	0.564	0.553	0.544	0.437	0.436
20	0.530	0.557	0.560	0.520	0.528	0.670	0.661	0.669	0.608	0.630
50	0.793	0.814	0.819	0.819	0.804	0.849	0.860	0.837	0.831	0.845

Table 10 shows the recall scores for the safe and the fast strategies when varying the number of features and the ratio of suspicious accounts in the dataset. The highest classification recall scores for both strategies are usually achieved when selecting the most important 25 or 50 variables. The ratio of suspicious accounts had a low impact on the recall score of the safe strategy and a moderate impact on the fast strategy. Similar to the baseline model, the safe strategy was better at reducing the number of false negatives, hence the higher recall scores of the strategy.

In Table 11, the change of the precision scores as the model parameters are varied can be observed. Selecting the best 25 features generally improved the strategies’ ability to reduce the number of false positives. Unlike the recall scores, however, reducing the class imbalance appears to significantly enhance the precision scores for both strategies. This is expected since we maintained the same number of suspicious accounts while increasing the number of normal accounts when reducing the ratio of suspicious accounts, due to the scarcity of suspicious accounts in the dataset provided to us. As such, an increasing number of normal observations was attributed to SOM neurons in the high risk category when a low suspicious accounts ratio was used in the dataset.

Furthermore, we used the Welch’s *t*-test (Welch, 1947) to investigate to what extent are the classification scores of the same strategy statistically significant when modifying the class ratios. To easily interpret the results of the significance test, we only used the scores of the experiments involving 25 selected features. In addition, we computed the statistical significance of a given strategy at once, since it is already expected that the strategies behave differently, thus, are already statistically significant. The *p*-values of the significance tests for the recall and precision scores are shown in Tables 12, 13.

**TABLE 12:** Recall scores’ *p*-values between different suspicious ratios.

Suspicious ratio (%)	Safe strategy			Fast strategy		
	Suspicious Ratio			Suspicious Ratio		
	10%	20%	50%	10%	20%	50%
5	0.299	0.549	0.457	0.587	0.059	0.378
10		0.564	0.609		0.049	0.609
20			0.879			0.114

**TABLE 13:** Precision scores’ *p*-values between different suspicious ratios.

Suspicious ratio (%)	Safe strategy			Fast strategy		
	Suspicious Ratio			Suspicious Ratio		
	10%	20%	50%	10%	20%	50%
5	0.000	0.000	0.000	0.024	0.000	0.000
10		0.000	0.000		0.007	0.000
20			0.000			0.000

It is evident from Table 12 that the recall scores of the safe strategy are not statistically significant as they are all greater than 0.05: the conventional *p*-value threshold of 5*%*. In the fast strategy, the recall scores are also statistically insignificant, with the exception being a suspicious ratio of 10% against 20%. Therefore, it can be inferred that varying the class ratio has a minimal impact on the ratio of false negatives of both strategies. In contrast, Table 13 reveals how the class imbalance greatly affects the change in the ratio of false positive classifications. Observing the outstandingly low *p*-values when comparing the class ratios demonstrate that the classification of normal accounts tend to improve when the dataset is characterised by a lower class imbalance. Hence, the significance test results indicate that our model produces a consistent classification of the suspicious accounts, while the increasing misclassification of normal accounts when reducing the proportion of suspicious accounts is inevitable due to the increased number of observations in our dataset.

## 6 Conclusion

In this work, we presented a method using self-organising maps to identify and detect suspicious accounts. In contrast to the other studies presented in the literature, we developed a model that detects money laundering activity in an imbalanced dataset while demonstrating robustness against the inadequately labelled data. The poor labelling is a general problem in money laundering datasets which stems from the fact that a remarkable proportion of money laundering transactions are undetected by conventional rule-based alert systems. Therefore, developing models based on supervised methods is not practical, as the class labels in transactional datasets do not necessarily capture the ground truths regarding whether a transaction was carried for funneling dirty money or not. To this end, we developed a framework based on SOMs to categorise the bank accounts in our dataset into three risk level groups. Our approach is adaptive, as we used the risk levels to propose two investigations strategies which can be employed by financial compliance teams in order to prioritise the investigation of suspicious accounts.

Evaluating the model demonstrated that self-organising maps tend to group the suspicious accounts together on the SOM grid, despite not reading the labels. The two proposed strategies allowed us to more evidently observe the false-negatives, false-positives dilemma that indeed exists in anti-money laundering practices. We highlight that our model presents a novel contribution to the literature by demonstrating its capability in detecting the majority of suspicious cases when choosing a safe investigation strategy, and reasonably reducing the number of false alerts when employing a less conservative strategy. In addition, unlike the classical black-box machine learning methods, SOMs enable the visualisation of samples along a low dimensional grid such that it is possible to observe the clusters of the grid and potentially categorise the accounts into more groups.

We note that while our framework presented a good detection of suspicious bank clients, our study is not without its limitations. First, the efficiency of the model drops when the there is a significant class imbalance in the dataset. The risk categorisation of our SOM is a semi-supervised task since we introduce a threshold based on the suspicious accounts ratio in the data. Consequently, while the model succeeds in clustering the suspicious observations together, many normal accounts are also attributed to these suspicious clusters, hence more time and resources spent on false alerts. Contrarily, some of the suspicious accounts are also classified as normal, which carries a much higher cost for financial institutions for failing to report suspicious activity. Secondly, due to the scarcity of suspected money laundering accounts, we used the binary labels to combine all suspicious accounts together. In practice, suspected money launderers are investigated differently based on a range of factors: corporate or individual accounts, daily or savings accounts, local or international transactions.

Future extensions to this work can include embedding more features such as account type, previous bankruptcies and account creation date, which might contribute to more distinction between normal and money laundering activity. In addition, we aim to obtain more data in order to use SOM for generating more clusters that can strengthen the understanding of the various underlying financial criminal behaviours. As a potential extension to this work, we plan to explore the impact of combining alternative unsupervised approaches such as growing neural gas (Fritzke et al., 1995) with supervised learning models for investigating money laundering accounts. Another promising direction for future works is incorporating tools commonly used in information retrieval systems such as the HITS algorithm (Kleinberg et al., 2011) for ranking the bank accounts based on suspicion level, which can help in prioritising the order by which clients are investigated.

## Data Availability

The data analyzed in this study is subject to the following licenses/restrictions: The data used for the purpose of this study was provided to us by our partner bank, DNB, and is subject to privacy and confidentiality agreements that prohibits us from publicly sharing it. Requests to access these datasets should be directed to Karl Aksel Festø, karl.aksel.festo@dnb.no.
